# Predisposition of Neonatal Maternal Separation to Visceral Hypersensitivity via Downregulation of Small-Conductance Calcium-Activated Potassium Channel Subtype 2 (SK2) in Mice

**DOI:** 10.1155/2020/8876230

**Published:** 2020-09-22

**Authors:** Ke Wu, Jing-hua Gao, Rong Hua, Xiao-han Peng, Hui Wang, Yong-mei Zhang

**Affiliations:** ^1^Jiangsu Province Key Laboratory of Anesthesiology, Xuzhou Medical University, Xuzhou, China; ^2^Emergency Department, The Affiliated Hospital of Xuzhou Medical University, Xuzhou, China

## Abstract

**Background:**

Visceral hypersensitivity is a common occurrence of gastrointestinal diseases such as irritable bowel syndrome (IBS), wherein early-life stress (ELS) may have a high predisposition to the development of visceral hypersensitivity in adulthood, with the specific underlying mechanism still elusive. Herein, we assessed the potential effect of small-conductance calcium-activated potassium channel subtype 2 (SK2) in the spinal dorsal horn (DH) on the pathogenesis of visceral hypersensitivity induced by maternal separation (MS) in mice.

**Methods:**

Neonatal mice were subjected to the MS paradigm, an established ELS model. In adulthood, the visceral pain threshold and the abdominal withdrawal reflex (AWR) were measured with an inflatable balloon. The elevated plus maze, open field test, sucrose preference test, and forced swim test were employed to evaluate the anxiety- and depression-like behaviors. The expression levels of SK2 in the spinal DH were determined by immunofluorescence and western blotting. The mRNA of SK2 and membrane palmitoylated protein 2 (MPP2) were determined by quantitative real-time polymerase chain reaction (qRT-PCR). Electrophysiology was applied to evaluate the neuronal firing rates and SK2 channel-mediated afterhyperpolarization current (*I*_AHP_). The interaction between MPP2 and SK2 was validated by coimmunoprecipitation.

**Results:**

In contrast to the naïve mice, ethological findings in MS mice revealed lowered visceral pain threshold, more evident anxiety- and depression-like behaviors, and downregulated expression of membrane SK2 protein and MPP2 protein. Moreover, electrophysiological results indicated increased neuronal firing rates and decreased *I*_AHP_ in the spinal DH neurons. Nonetheless, intrathecal injection of the SK2 channel activator 1-ethyl-2-benzimidazolinone (1-EBIO) in MS mice could reverse the electrophysiological alterations and elevate the visceral pain threshold. In the naïve mice, administration of the SK2 channel blocker apamin abated *I*_AHP_ and elevated spontaneous neuronal firing rates in the spinal DH neurons, reducing the visceral pain threshold. Finally, disruption of the MPP2 expression by small interfering RNA (siRNA) could amplify visceral hypersensitivity in naïve mice.

**Conclusions:**

ELS-induced visceral pain and visceral hypersensitivity are associated with the underfunction of SK2 channels in the spinal DH.

## 1. Introduction

Irritable bowel syndrome (IBS) is a prevalent functional gastrointestinal disorder mainly characterized by visceral hypersensitivity, of which chronic visceral pain is one of the most common symptoms [1]. Epidemiological studies suggest that the IBS prevalence reached approximately 11% worldwide, and about 94% of IBS patients presented with visceral hypersensitivity. However, with respect to the specific mechanism of visceral hypersensitivity, the complex etiological factors such as changes of immune and neuroendocrine systems, psychological and physical stresses, and genetic and early life influences still await further elucidation [2–4].

Our prior report confirmed that early-life stresses (ELS) such as colorectal distension (CRD) and maternal separation (MS) can cause visceral hypersensitivity in rats in adulthood [5–7]. In addition, clinical and preclinical data indicate that ELS is an important predisposing factor of long-term hyperalgesia [8]. Animal studies have shown that early life is a critical period for the normal development of individuals, during which physical and psychological stresses can lead to neuroendocrine changes and exert effects on the signal pathways involved in neuroplasticity regulation, accompanied by corresponding alterations of neurobehaviors, including learning and memory and anxiety-like and depression-like behaviors [9, 10]. Moreover, ELS can reportedly exaggerate stress-induced visceral hypersensitivity via action on the hypothalamic-pituitary-adrenal (HPA) axis, the autonomic nervous system (ANS), and the epigenetic modification [11, 12]. Clinical evidence demonstrates that ELS can induce major consequences in the nervous system and plays a pivotal role in the pathophysiology of a variety of disorders, such as IBS, major depressive disorder (MDD), and posttraumatic stress disorder (PTSD), all of which can severely impede behavioral and cognitive well-being of humans [13–15]. However, a number of studies indicate that ELS (physical or psychological or both) can lead to different long-term behavioral and physiological alterations in adulthood via activation of diverse neural networks [16–18]. Of note, distinctive to the physical stress, which exerts earlier but relatively mild impact, the psychological stress renders later but more severe consequences [19]. Given the important role of early psychological life stress in predisposing individuals to physical and mental health disorders in adulthood, we adopted the scheme of MS to induce visceral hypersensitivity [20].

Visceral sensory signals are transmitted to various corticolimbic structures via parasympathetic afferent and sympathetic afferent pathways, in which the dorsal horn (DH) of the spinal cord plays a vital role via reception of visceral information from the dorsal root ganglia and relaying to the neuromatrix of the pain [21–23]. In addition, the hyperexcitability of visceral nociceptive neurons in the spinal cord DH is involved in visceral hypersensitivity [24–26]. Hence, the modulation of the excitability of these nociceptive neurons has invited intriguing approaches to the therapeutics of visceral hypersensitivity. Small-conductance calcium-activated potassium (SK) channels in dendritic spines are reportedly able to control the excitability of neuronal cells via the regulation of synaptic transmission [27].

SK channels constitute a distinct subfamily of potassium channels, which are sensitive to intracellular calcium ions with no voltage dependence [28]. SK channels are the modulators of neuronal excitability, involved in the mediation of afterhyperpolarization current (*I*_AHP_) and the regulation of neuronal firing rates [29, 30]. Immunohistochemical findings have validated that the SK channels are widely expressed throughout the brain [31], spinal dorsal root ganglia, and spinal DH neurons [32]. With respect to their physiological and pharmacological characterization, the SK channels are categorized into three subtypes: SK1, SK2, and SK3 channels [33], with SK2 channels almost entirely located in the superficial layer of the spinal DH [32]. SK channels in the spinal DH are involved in the modulation of nociception, as illustrated by the SK channel opener 1-EBIO in mitigation of the thermal-induced nociception behavior by reducing spike discharges and increasing *I*_AHP_ amplitudes [34]. In addition, activation of SK channels in the spinal cord can also ameliorate mechanical hypersensitivity in a rat model of inflammatory pain [35]. Conversely, intraplantar injection of a specific SK channel inhibitor induces mechanical allodynia and heat hyperalgesia in naïve rats [36]. Furthermore, the localization of SK2 channels on the cell surface membrane can be dynamically governed by the anterograde and retrograde trafficking [37]. Recently, a novel synaptic scaffold MPP2 has been reported to be responsible for locating the SK2 channel at the synapses, which allows SK2 channels to contribute to long-term potentiation (LTP) and synaptic plasticity fortification [38].

In the present study, we examined the variation of the SK2 protein expression and SK2 channel activity with western blotting and electrophysiological recording in mice presenting with MS-induced visceral hypersensitivity. The interaction between MPP2 and SK2 was determined by coimmunoprecipitation.

## 2. Methods and Materials

### 2.1. Animals

C57BL/6J mice were provided by the Experimental Animal Center of Xuzhou Medical University (Xuzhou, China), with one male mouse cohabiting with two female mice to facilitate reproduction, with controlled temperature and humidity (22°C and 50%) and *ad libitum* access to food and water. Model mice were exposed to maternal separation protocol, and only males were used for the experiments. All procedures were conducted in accordance with the NIH Guide for the Care and Use of Laboratory Animals (2011) and approved by the Institutional Animal Care and Use Committee at Xuzhou Medical University.

### 2.2. Maternal Separation Protocol

**Figure 1:**
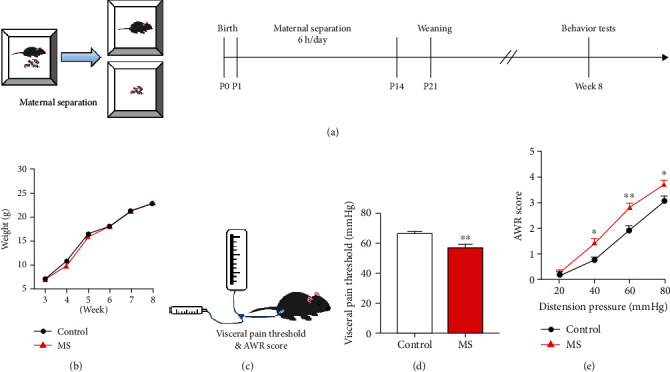
Mice undergoing neonatal MS exhibited visceral hypersensitivity. (a) Experimental paradigm of the maternal separation. (b) There was an insignificant difference in body weight between control and MS mice within 5 weeks after weaning (two-way ANOVA test, *F* (1, 22) = 0.51, *P* = 0.482, *n* = 12 per group). (c) Schema of assessment of abdominal withdrawal reflex (AWR) and visceral pain threshold. (d) The visceral pain threshold of mice experiencing neonatal MS presented a significant decline (*t*-test, *t* = 3.11, *P* < 0.01, *n* = 12 per group). (e) The AWR score displayed a significant increase in MS mice subjected to pressure stimuli at 40 mmHg (Bonferroni *post hoc* test, *P* < 0.05), 60 mmHg (Bonferroni *post hoc* test, *P* < 0.01), and 80 mmHg (Bonferroni *post hoc* test, *P* < 0.05), respectively (two-way ANOVA test, *F* (1, 22) = 9.82, *P* < 0.01, *n* = 12 per group). Data are expressed as mean ± SEM; ^∗^*P* < 0.05 and ^∗∗^*P* < 0.01.

Maternal separation of pups from their dams was sustained for 6 h daily (8:00-11:00 A.M. and 2:00-5:00 P.M.), as from postnatal day 2 (P1) to day 15 (P14), with the pups of the same brood isolated in one cage (15 × 20 cm) maintained at 32 ± 0.5°C (P1-P5) or 30 ± 0.5°C (P6-P14). At the end of separation of 3 h, pups were returned to their home cages (Figure 1(a)), whereas control mice were standard facility reared [39, 40].

### 2.3. Reagents

The reagents were as follows: rabbit anti-SK2/K_Ca_2.2 polyclonal antibody (APC-028, Alomone Labs, Israel), Alexa Fluor 594 donkey anti-rabbit IgG (H+L) (A21207, Thermo Fisher Scientific, Waltham, MA, USA), mouse anti-GAPDH mAb (AC001, ABclonal, Waltham, MA, USA), mouse anti-*β*-actin mAb (sc-47778, Santa Cruz Biotechnology, USA), HRP-labeled goat anti-rabbit IgG (H+L) (A0208, Beyotime, China), HRP-labeled goat anti-mouse IgG (H+L) (A0216, Beyotime), alkaline phosphatase goat anti-rabbit IgG (ZB-2308, Beyotime), BCA protein assay kit (P0012, Beyotime), sodium dodecyl sulfate- (SDS-) polyacrylamide gel electrophoresis (PAGE) sample loading buffer (P0015, Beyotime), BCIP/NBT alkaline phosphatase color development kit (C3206, Beyotime), BeyoECL Moon kit (P0018FFT, Beyotime), and Syn-PER™ Synaptic Protein Extraction Reagent (#87793, Thermo Fisher Scientific, Waltham, MA, USA).

### 2.4. Assessment of Abdominal Withdrawal Reflex (AWR) and Visceral Pain Threshold

Visceral sensitivity was assessed by AWR and visceral pain threshold [7, 41]. Mice were displaced in transparent plastic boxes (10 cm × 10 cm × 5 cm) on an elevated plexiglass platform, allowed for habituation for 15-20 min. We conducted graded distension by rapid inflation of a balloon (2 cm × 1.5 cm) inside the colorectal region to a distension pressure (20, 40, 60, or 80 mmHg) for a duration of 20 s at an interval of 2-3 min (Figure 1(c)). The AWR was scored as follows: 0, absence of behavioral response; 1, brief head movement followed by immobility; 2, contraction of the abdominal muscles; 3, elevation of the abdomen; and 4, body arcing and elevation of the pelvic structures. The visceral pain threshold was defined by the stimulus intensity whereby to evoke a visible contraction of the abdominal wall. For accuracy, distension was conducted in triplicate to calculate an average. In the process of behavioral test, the technicians were blinded to animal grouping. A technician performed the inflation via a syringe at a steady speed, with another technician observing the changes of abdominal movement and recording the readings on the sphygmomanometer.

### 2.5. Open Field Test

Open field test was performed to assess anxiety-related behavior. The apparatus is a square-shaped enclosure, consisting of a black wooden floor (50 cm × 50 cm) surrounded by walls (50 cm in height). The test commenced with mouse displacement at the corner of the open field and allowed for free exploration within 5 min, with the times of line crossings, duration in the center zone (16.5 × 16.5 cm), and times of entry into the central zone recorded with the ANY-maze video tracking software. At the end of each mouse experiment, the whole area was cleansed with 70% alcohol to remove the odor.

### 2.6. Elevated Plus Maze

The elevated plus maze was also employed to detect anxiety-like behavior. The apparatus consists of two opposing closed arms and open arms (30 cm × 10 cm) with walls (10 cm in height) of the same dimensions and an arena (10 cm × 10 cm) at the intersection of the open arms and closed arms. During the 5 min test, the mouse was first placed on the arena, facing the closed arm. The observation indexes included the following: the time spent in the open arms and the numbers of entry into the open arms. Entry into the open arms was defined as all four paws within the open arms area, with an ANY-maze video tracking system for camera control and data recording, with the same cleansing procedures of four arms as described afore.

### 2.7. Sucrose Preference Test

Prior to testing, mice were supplied with two bottles of tap water for the first day, followed by replacement with the 1% sucrose solution for the second day. On the third day, mice were deprived of water. On the fourth day, the mice were provided with two bottles filled with 1% sucrose solution and tap water each. Finally, sucrose preference was calculated as the consumption volume of sucrose solution over total volume of liquid intake.

### 2.8. Forced Swim Test

Each mouse was gently immerged in water at 25°C and 30 cm in depth (beyond the reach of bottom) in a plastic cylinder for 1 min adaption, with the immobility duration within 5 min recorded. Immobility is defined as the maintenance of an immobile posture in one place, or minimum paw movement necessary to keep its nose and eyes above water, with prolonged immobility duration deemed as “behavioral despair.”

### 2.9. Intrathecal Catheter Implantation

With the mice anesthetized under 2% pentobarbital sodium (40 mg/kg, i.p.), a longitudinal dorsal incision (approximately 1 cm) was made through the skin and muscle, with the L5-L6 vertebrae fully exposed. PE catheter (0.23 mm OD × 0.09 mm ID) containing 0.9% sterile saline was inserted between L5 and L6. Sterile saline (1 *μ*L) was gently injected into the subarachnoid space to ensure that the catheter was unobstructed. The outside end of the catheter was sealed, and the catheter was secured to the adjacent tissues. With antiseptic treatment, mice were allowed for recovery for 5 days. 1-EBIO (30 *μ*g), apamin (0.5 ng), and small interfering RNA (siRNA) targeting MPP2 (5 *μ*g) were administered in a volume of 5 *μ*L via a microsyringe infusion pump (KDS Scientific, USA) loaded with a 10 *μ*L mL Hamilton microsyringe.

### 2.10. Electrophysiology

For *in vitro* recordings, 45 min after the last behavior test, mice underwent transcardial perfusion under deep anesthesia; transverse sections of the lower lumbar and upper sacral segment (L4-S4) were sliced at the thickness of 300 *μ*m on a vibratome (VT1200S, Leica, Germany) in an ice-cold high-sucrose cutting solution (mM) comprising 80 NaCl, 4.5 MgSO_4_, 3.5 KCl, 0.5 CaCl_2_, 1.25 NaH_2_PO_4_, 90 sucrose, 25 NaHCO_3_, and 10 glucose; artificial cerebral spinal fluid (ACSF) (mM): 126 NaCl, 1.2 NaH_2_PO_4_, 2.5 KCl, 1.2 MgSO_4_, 26 NaHCO_3_, 10 glucose, and 2.4 CaCl_2_. All solutions were saturated with 95% O_2_ and 5% CO_2_. The slices were transferred to the ACSF at room temperature (r/t) for 1 h after incubation in high-sucrose cutting solution at 34°C for 15-20 min. Patch-clamp recordings were performed in DH laminae I-II neurons with standard wall-borosilicate glass pipette (BF-150-86-10, SUTTER, USA) filled with an internal solution (mM) consisting of 10 phosphocreatine-Tris, 10 HEPES, 2 ATP-Mg, 10 EGTA, 0.5 GTP-Na, 115 K gluconate, 20 KCl, and 1.5 MgCl_2_; pH was adjusted to 7.25 with KOH (280-290 mOsm). The pipette resistance was set at 5-10 M*Ω*. For whole-cell recording, DH neurons were maintained in a voltage clamp at a holding potential of -60 mV and 100 ms depolarizing pulse to 60 mV, which could evoke an outward current to measure SK currents. Cell-attached recordings were performed in the voltage clamp with the current set at 0 pA on a MultiClamp 700B amplifier (Axon Instruments, Union City, CA, USA). The data were acquired and analyzed with Clampex and Clampfit 10 (Axon Instruments, San Jose, CA, USA).

### 2.11. Western Blotting

45 min after the last behavior test, the mice were sacrificed, with the spinal cord at the lower lumbar and upper sacral segments (L4-S4) isolated on ice and placed in an Eppendorf (EP) tube and thereafter stored in the icy environment. The lysate Syn-PER™ Synaptic Protein Extraction Reagent (1 mL/100 mg) and the phosphatase inhibitor phenylmethylsulfonyl fluoride (PMSF) were successively added, followed by homogenization and centrifugation at 8000 rpm for 10 min at 4°C, with 80 *μ*L supernatant (whole-cell lysis) sampled thereafter. Then, the supernatant was centrifuged at 12000 rpm for 20 min, with the cracking buffer (20 *μ*L) added to the particles (membrane section). The BCA method was employed to detect and trim the protein concentration after lysis. Equal amounts of protein were separated by SDS-PAGE gels and transferred onto the PVDF membrane. After blockade with 5% nonfat milk for 2 h at r/t, the PVDF membranes were incubated with anti-SK2 (1 : 200) and anti-GAPDH (1 : 1000) primary antibodies at 4°C overnight. After lavage in Tris-buffered saline with Tween (TBST), the PVDF membrane was incubated with HRP-conjugated secondary antibody (1 : 1000) for 2 h at r/t. Western blotting analyses were performed with ImageJ software (NIH, USA).

### 2.12. Immunofluorescence

Mice were transcardially perfused with 0.9% saline (10 mL/10 g), followed by 4% polyformaldehyde in phosphate buffer (10 mL/10 g) after deep anesthesia. The spinal cord (L4-S4) was isolated and fixed in 4% polyformaldehyde for 24 h and equilibrated in 30% sucrose solution prior to slice preparation at a thickness of 30 *μ*m with a cryostat (CM1800, Leica, Germany). Selected slices were thrice rinsed with PBS for 10 min and blocked with 10% donkey serum at r/t for 2 h before incubation with anti-SK2 (1 : 150) at 4°C for 24 h. Subsequently, the slices were lavaged thrice and incubated with Alexa Fluor 594 (1 : 200) at r/t for 2 h. Images were obtained with the use of a confocal laser microscope (FV1000, Olympus, Japan).

### 2.13. Quantitative Real-Time Polymerase Chain Reaction (qRT-PCR)

45 min after the last behavior test, mice were sacrificed under pentobarbital sodium. The spinal cord was quickly removed on ice, with the spinal cord at lower lumbar and upper sacral segment (L4-S4) dissected and total RNA extracted with Spin Column Animal Total RNA Purification Kit (Shenggong, China) as per standard protocols. RNA preparations were reverse transcribed to generate cDNA with FastKing RT Kit (TIANGEN, China). The cDNA products served as templates for real-time PCR analysis to observe the SK2 expression with the SK2-specific primers (Shenggong, China). The primers applied to amplify SK2 were 5′-CTGCTTGCTTACTGGAATCATG-3′ (forward) and 5′-CATCATGAAATTGTGCACATGC-3′ (reverse). Sense and antisense primers were located on different exons to avoid false-positive results due to genomic DNA contamination. PCRs were performed on LightCycler 480 System via fluorescent SYBR Green technology (Applied Biosystems, USA). Reaction protocols were in the following schema: 1 min at 95°C for enzyme activation followed by 40 cycles of 10 s at 95°C and 30 s at 60°C and 30 s at 72°C, followed by 95°C for 5 s and 60°C for 60 s. Melting curve analysis was performed to check the specificity of the amplification products. All reactions contained the same amount of cDNA. The relative expression ratio of SK2 mRNA was normalized to GAPDH gene expression using the *Δ*Ct method (2^-*ΔΔ*Ct^).

### 2.14. Coimmunoprecipitation

The lysate was centrifuged at 13000 rpm for 20 min at 4°C. The supernatant was incubated with the indicated anti-SK2 for 30 min, and then, 20 *μ*L of protein A/G agarose beads (Thermo Scientific, USA) was added for incubation overnight at 4°C with rotation. The beads were rinsed in triplicate in immunoprecipitation lysate. Proteins were eluted with 20 *μ*L of 2x SDS sample buffer at 37°C. Bound and eluted proteins were subsequently separated by SDS-PAGE and transferred to the PVDF membrane. After blockade with 5% skim milk for 2 h, the membrane was probed with the anti-MPP2 overnight at 4°C. HRP-conjugated secondary antibody was applied for 45 min. Blot analyses were performed with ImageJ software (NIH, USA).

### 2.15. Statistical Analysis

Data are expressed as mean ± SEM. For independent samples, Student's *t*-test and two-way analysis of variance (ANOVA) test followed by Bonferroni's *post hoc* test or S-N-K multiple comparisons were employed. All statistical analyses were conducted with the SPSS 19.0 (IBM, USA) software. *P* < 0.05 was considered statistically significant.

## 3. Results

### 3.1. ELS Induced Visceral Hypersensitivity in Mice Undergoing Neonatal MS

The canonical model of MS serves as a preclinical model of ELS. Murine pups were separated from their dams for 6 h daily as of postnatal day 2 (P1) to day 15 (P14). At week 8 (reaching adulthood), AWR and visceral pain threshold were measured (Figures 1(a) and 1(c)). There was an insignificant difference in body weight between control and MS mice within 5 weeks after weaning (*n* = 12, *P* = 0.482, Figure 1(b)), suggesting that mice did not suffer from malnutrition due to MS. Neonatal MS altered the visceral pain threshold and AWR score. Mice experiencing neonatal MS presented with lower pain threshold compared with control mice (*n* = 12, *P* < 0.01, Figure 1(d)). Consistently, MS mice presented enhanced AWR score compared to control mice. We further analyzed the statistical difference in each distension pressure and identified that neonatal MS increased AWR score at pressure stimuli at 40 mmHg (*P* < 0.05), 60 mmHg (*P* < 0.01), and 80 mmHg (*P* < 0.05), respectively (*n* = 12, Figure 1(e)).

### 3.2. Mice with Neonatal MS Presented Anxiety- and Depression-Like Behaviors

**Figure 2:**
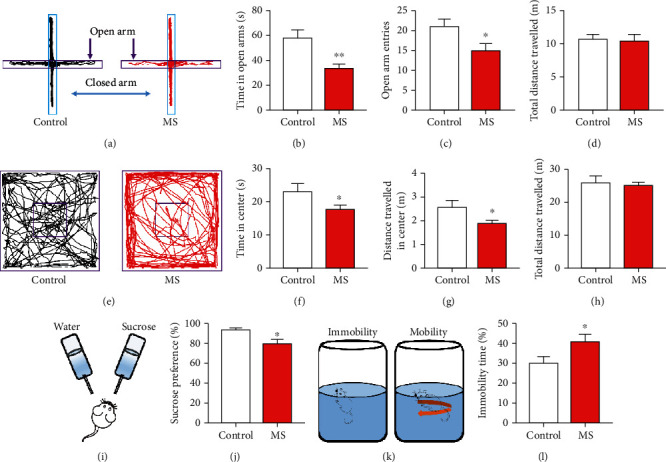
Mice experiencing neonatal MS showed anxiety- and depression-like behaviors. (a) Representative activity traces of control and MS mice in the elevated plus maze. (b–d) The duration spent in the open arms (*t*-test, *t* = 3.06, *P* < 0.01) and the times of entry into the open arms (*t*-test, *t* = 2.48, *P* < 0.05) were significantly reduced in the MS mice compared with the control mice (*n* = 8 per group), with no significant difference in the total distances travelled between the control and MS mice (*t*-test, *t* = 0.28, *P* = 0.783, *n* = 8 per group). (e) Schema of the open field and the representative activity traces in control and MS mice. (f–h) The duration in the central area (*t*-test, *t* = 2.13, *P* < 0.05) and the courses in the central area (*t*-test, *t* = 2.28, *P* < 0.05) were significantly reduced in the MS group compared with the control group (*n* = 8 per group); similarly, there was an insignificant difference in the total distances travelled between the control and MS mice (*t*-test, *t* = 0.46, *P* = 0.653, *n* = 8 per group). (i) Schematic diagram of the sucrose preference test. (j) The percentage of the sucrose intake volume over the total fluid intake volume in the MS mice was significantly smaller than that in the control mice (*t*-test, *t* = 2.57, *P* < 0.05, *n* = 8 per group). (k) Schematic representation of mobility and immobility in the forced swim test. (l) The percentage of immobility duration over the total time in the MS mice was significantly greater than that in the control mice (*t*-test, *t* = 2.36, *P* < 0.05, *n* = 8 per group). Data are expressed as mean ± SEM; ^∗^*P* < 0.05 and ^∗∗^*P* < 0.01.

The elevated plus maze and open field test serve to investigate the effect of MS on anxiety-like behaviors. As demonstrated by representative traces in elevated plus maze in Figure 2(a), compared with the control mice, MS mice were markedly inactive in the open arms of the maze as evidenced by decreased time (*n* = 8, *P* < 0.01, Figure 2(b)) and times of total entry in the open arms (*n* = 8, *P* < 0.05, Figure 2(c)). The same total distance of travel between the control and MS mice authenticated the intactness of motor ability (*n* = 8, *P* = 0.783, Figure 2(d)). Mice with neonatal MS exhibited significantly diminished duration in the central area (*n* = 8, *P* < 0.05, Figures 2(e) and 2(f)) and distance covered in the central area (*n* = 8, *P* < 0.05, Figures 2(e) and 2(g)) compared with the control group, with no significant difference in the total distance of travel between the control and MS mice (*n* = 8, *P* = 0.653, Figures 2(e) and 2(h)). The results of the elevated plus maze and open field test indicated MS mice had attenuated exploration attempt and remained close to the walls compared with the control mice. To verify whether MS mice exhibited depression-like behaviors, the reward-based sucrose preference test and forced swim test were involved. Compared to the MS mice, the control mice had a significant preference for sucrose (*n* = 8, *P* < 0.05, Figures 2(i) and 2(j)), indicating the presence of depression-like behaviors in the MS mice. In addition, we measured the perseverance of mice to identify a depressive phenotype, via the forced swim test. The results revealed that MS mice exhibited increased percentage of immobility duration in the total time compared with the control mice (*n* = 8, *P* < 0.05, Figures 2(k) and 2(l)). Thus, these data indicated that mice experiencing neonatal MS presented the anxiety- and depression-like behaviors.

### 3.3. The Decline of SK2 Protein in Membrane Fraction, SK2 mRNA, and *I*_AHP_ in Spinal DH in Mice Experiencing Neonatal MS

**Figure 3:**
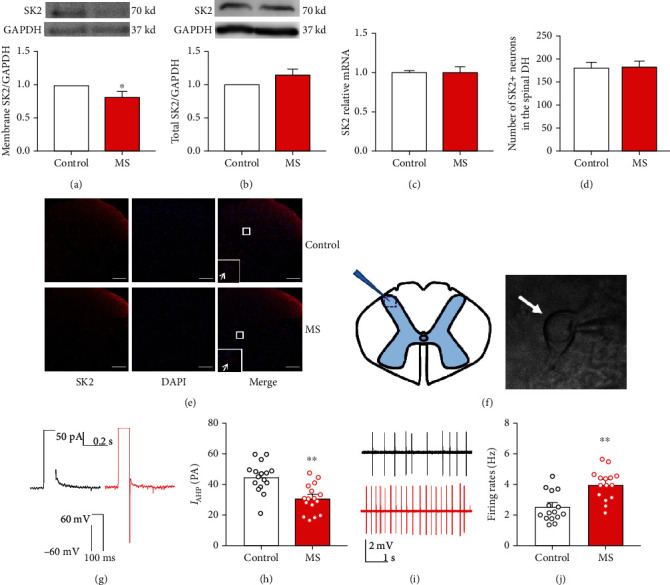
SK2 protein expressions in the membrane fraction, SK2 mRNA, and *I*_AHP_ were reduced in mice with neonatal MS. (a) Spinal SK2 protein in the membrane fraction presented a significant decrease in mice with the neonatal MS versus control mice (*t*-test, *t* = 2.77, *P* < 0.05, *n* = 6 per group). (b) There was no difference in total spinal SK2 channel protein in mice with the neonatal MS versus control mice (*t*-test, *t* = 1.53, *P* = 0.157, *n* = 6 per group). (c) Consistently, there was also no difference in total spinal SK2 mRNA in mice with the neonatal MS versus control mice (*t*-test, *t* = 0.16, *P* = 0.877, *n* = 9 per group). (d) There was no difference in the number of SK2+ neurons in the spinal dorsal horn between the two groups (*t*-test, *t* = 0.29, *P* = 0.783, *n* = 4 per group). (e) SK2 channel immunostaining, scale bar = 100 *μ*m. (f) Schema of patch-clamp recording. (g, h) The average peak amplitude of *I*_AHP_ in the neonatal MS mice was significantly reduced versus that in the control mice (*t*-test, *t* = 4.18, *P* < 0.01, *n* = 16 neurons/5 mice per group). (i, j) The spontaneous neuronal firing rates in the spinal DH increased significantly in the neonatal MS mice versus the control mice (*t*-test, *t* = 4.19, *P* < 0.01, *n* = 15 neurons/5 mice per group). Data are expressed as mean ± SEM, ^∗∗^*P* < 0.01.

Western blotting data showed that the spinal SK2 protein in the membrane fraction presented a significant decrease in the MS mice compared with the control mice (*n* = 6, *P* < 0.05, Figure 3(a)). However, there was no difference in the total spinal SK2 protein between the two groups (*n* = 6, *P* = 0.157, Figure 3(b)). Consistently, the same was also true of the total spinal SK2 mRNA (*n* = 9, *P* = 0.877, Figure 3(c)). The expression of SK2 showed that the SK2 channel is widely expressed across the spinal dorsal horn in either the control mice or mice with neonatal MS, and there was no difference in the number of SK2+ neurons in the spinal dorsal horn between the two groups (*n* = 4, *P* = 0.783, Figures 3(d) and 3(e)). Mice with neonatal MS presented lower *I*_AHP_ compared to mice without neonatal MS. The average peak amplitude of *I*_AHP_ was 45.35 ± 2.49 pA and 31.13 ± 2.32 pA in the control and MS mice, respectively (*n* = 16 neurons/5 mice, *P* < 0.01, Figures 3(g) and 3(h)). Consistently, mice with neonatal MS presented an increase in spontaneous neuronal firing rates versus control mice (2.54 ± 0.25 vs. 4.03 ± 0.26, *n* = 15 neurons/5 mice, *P* < 0.01, Figures 3(i) and 3(j)). The above results suggested that the function of the SK2 channel in MS mice was mitigated due to the experience of ELS, which led to increased neuronal activity in the spinal DH and ultimately visceral hypersensitivity.

### 3.4. Effects of SK2 Channel Blocker Apamin on *I*_AHP_, Spontaneous Neuronal Firing Rates, and Visceral Pain Threshold

**Figure 4:**
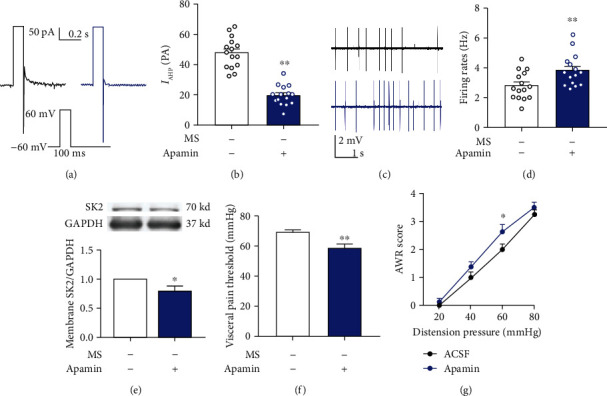
Effects of the SK2 channel blocker apamin on *I*_AHP_, spontaneous neuronal firing rates, SK2 protein in membrane fraction, and pain threshold. (a, b) Apamin injection decreased the amplitude of *I*_AHP_ in naïve mice (*t*-test, *t* = 8.97, *P* < 0.01, *n* = 15 neurons/5 mice per group). (c, d) Apamin injection increased the neuronal firing rates in the spinal DH of naïve mice (*t*-test, *t* = 2.82, *P* < 0.01, *n* = 15 neurons/5 mice per group). (e) Apamin injection resulted in the decrease of the spinal SK2 channel in membrane fraction in the naïve mice (*t*-test, *t* = 2.70, *P* < 0.05, *n* = 6 per group). (f) Apamin administration caused the decrease of the pain threshold in naïve mice (*t*-test, *t* = 3.00, *P* < 0.01, *n* = 8 per group). (g) Naïve mice with intrathecal injection of apamin exhibited increased AWR score in response to the distension pressure at 60 mmHg (Bonferroni *post hoc* test, *P* < 0.05) (two-way ANOVA test, *F* (1, 14) = 3.82, *P* = 0.07, *n* = 8 per group). Data are expressed as mean ± SEM; ^∗^*P* < 0.05 and ^∗∗^*P* < 0.01.

SK2 channel blocker apamin was added into the ACSF to reach the desired concentration (100 nM) which decreased *I*_AHP_ in mice without neonatal MS versus the control group (47.86 ± 2.65 vs. 19.65 ± 1.70, *n* = 15 neurons/5 mice, *P* < 0.01, Figures 4(a) and 4(b)) and increased the neuron firing rates (2.81 ± 0.24 vs. 3.85 ± 0.28, *n* = 15 neurons/5 mice, *P* < 0.01, Figures 4(c) and 4(d)). Western blotting data showed that the spinal SK2 protein in the membrane fraction presented a significant decrease in mice receiving apamin (*n* = 6, *P* < 0.05, Figure 4(e)). AWR and visceral pain threshold were measured 2 h after intrathecal administration of apamin (0.5 ng/5 *μ*L), and the results showed that apamin administration contributed to the decrease in visceral pain threshold (*n* = 8, *P* < 0.01, Figure 4(f)). Subsequent to apamin administration, naïve mice presented increased AWR score with pressure stimulus at 60 mmHg versus the ACSF control group (*n* = 8, *P* < 0.05, Figure 4(g)).

### 3.5. Effects of SK2 Channel Blocker Apamin on Anxiety- and Depression-Like Behaviors

**Figure 5:**
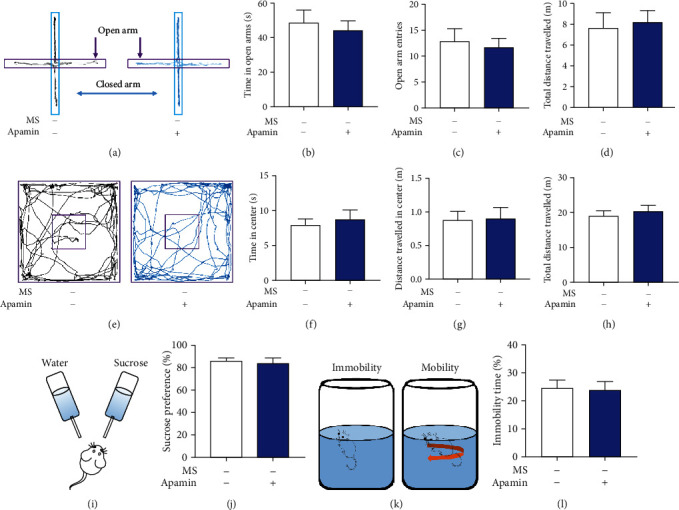
Effects of SK2 channel blocker apamin on anxiety- and depression-like behaviors. (a) Representative activity traces of control mice and mice with apamin injection in the elevated plus maze. (b–d) There was an insignificant difference in the duration spent in the open arms (*t*-test, *t* = 0.46, *P* = 0.657), the times of entry into the open arms (*t*-test, *t* = 0.36, *P* = 0.725), and the total distances travelled (*t*-test, *t* = 0.29, *P* = 0.777) between the two groups (*n* = 8 per group). (e) Schema of the open field and the representative activity traces during an open field test in control mice and mice with apamin injection. (f–h) There was an insignificant difference in the duration in the arena (*t*-test, *t* = 0.47, *P* = 0.645), the courses in the arena (*t*-test, *t* = 0.09, *P* = 0.933), and the total distances travelled (*t*-test, *t* = 0.57, *P* = 0.576) between the two groups (*n* = 8 per group). (i) Schema of the sucrose preference test. (j) The percentage of the sucrose intake volume over the total fluid intake volume in mice with apamin injection was consistent with that in control mice (*t*-test, *t* = 0.42, *P* = 0.679, *n* = 8 per group). (k) Schematic representation of mobility and immobility in the forced swim test. (l) The percentage of immobility duration over the total time in mice with apamin injection was consistent with that in control mice (*t*-test, *t* = 0.16, *P* = 0.877, *n* = 8 per group). Data are expressed as mean ± SEM.

Behavioral tests were measured 2 h after intrathecal administration of apamin (0.5 ng/5 *μ*L), and the results showed that apamin administration did not affect the anxiety-like behavior of mice. The duration spent in the open arms (*n* = 8, *P* = 0.657, Figures 5(a) and 5(b)), the times of entry into the open arms (*n* = 8, *P* = 0.725, Figures 5(a) and 5(c)), the duration in the arena (*n* = 8, *P* = 0.645, Figures 5(e) and 5(f)), and the courses in the arena (*n* = 8, *P* = 0.933, Figures 5(e) and 5(g)) showed insignificant differences between the control mice and mice with apamin injection. Likewise, apamin administration did not affect the depression-like behavior as evidenced by the same preference for sucrose (*n* = 8, *P* = 0.679, Figures 5(i) and 5(j)) and immobility duration (*n* = 8, *P* = 0.877, Figures 5(k) and 5(l)) between the two groups.

### 3.6. Effects of SK2 Channel Activator 1-EBIO on Visceral Hypersensitivity due to Neonatal MS in Mice

**Figure 6:**
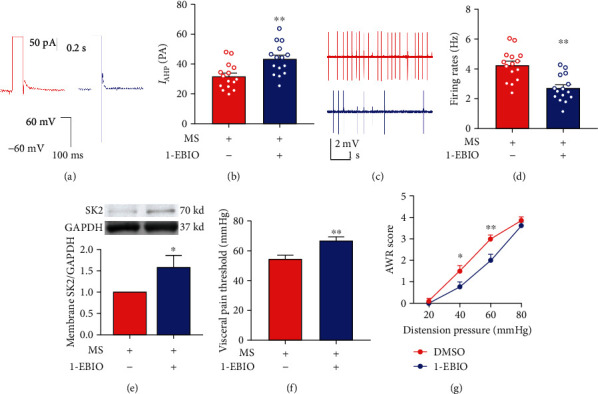
Effects of SK2 activator 1-EBIO on *I*_AHP_, spontaneous neuronal firing rates, SK2 protein in membrane fraction, and visceral pain threshold. (a, b) 1-EBIO administration increased the amplitude of *I*_AHP_ in mice with neonatal MS (*t*-test, *t* = 3.30, *P* < 0.01, *n* = 15 neurons/5 mice per group). (c, d) 1-EBIO administration decreased the neuronal firing rates in the spinal DH of mice with neonatal MS (*t*-test, *t* = 4.38, *P* < 0.01, *n* = 15 neurons/5 mice per group). (e) 1-EBIO administration caused the increase of the spinal SK2 channel in membrane fraction in MS mice (*t*-test, *t* = 2.43, *P* < 0.05, *n* = 6 per group). (f) 1-EBIO administration effectively reversed the decrease of visceral pain threshold due to neonatal MS (*t*-test, *t* = 3.02, *P* < 0.01, *n* = 8 per group). (g) MS mice with intrathecal injection of 1-EBIO exhibited decreased AWR scores in response to the distension pressures at 40 mmHg (Bonferroni *post hoc* test, *P* < 0.05) and 60 mmHg (Bonferroni *post hoc* test, *P* < 0.01) (two-way ANOVA test, *F* (1, 14) = 5.76, *P* < 0.05, *n* = 8 per group). Data are expressed as mean ± SEM; ^∗^*P* < 0.05 and ^∗∗^*P* < 0.01.

SK2 channel activator 1-EBIO was added into the ACSF to reach the desired concentration (100 *μ*M). 1-EBIO elevated *I*_AHP_ (31.81 ± 2.24 vs. 43.41 ± 2.71, *n* = 15 neurons/5 mice, *P* < 0.01, Figures 6(a) and 6(b)) and decreased the neuron firing rates (4.29 ± 0.27 vs. 2.73 ± 0.23, *n* = 15 neurons/5 mice, *P* < 0.01, Figures 6(c) and 6(d)) in the spinal DH neurons. Western blotting data showed that the spinal SK2 protein in the membrane fraction presented a significant increase in mice receiving 1-EBIO (*n* = 6, *P* < 0.05, Figure 6(e)). Mice underwent intrathecal administration of 1-EBIO (30 *μ*g/5 *μ*L) 2 h prior to behavioral tests. 1-EBIO administration reversed the decline in the visceral pain threshold in mice experiencing neonatal MS (*n* = 8, *P* < 0.01; Figure 6(f)). Consistently, subsequent to 1-EBIO administration, MS mice presented decreased AWR score versus dimethyl sulfoxide (DMSO) control group. With the incremental stimuli of distension pressures, MS mice exhibited decreased AWR scores at 40 mmHg (*P* < 0.05) and 60 mmHg (*P* < 0.01) after the administration of 1-EBIO (*n* = 8, Figure 6(g)).

### 3.7. Effects of SK2 Channel Activator 1-EBIO on Anxiety- and Depression-Like Behaviors

**Figure 7:**
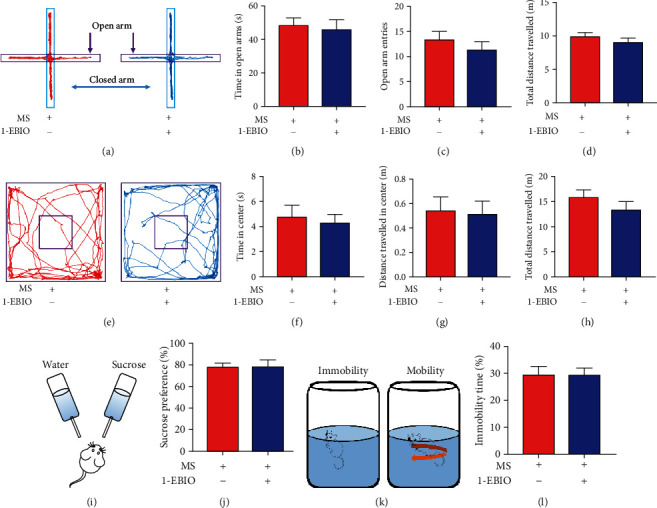
Effects of SK2 activator 1-EBIO on anxiety- and depression-like behaviors. (a) Representative activity traces of control mice and mice with 1-EBIO injection in the elevated plus maze. (b–d) There was an insignificant difference in the duration spent in the open arms (*t*-test, *t* = 0.35, *P* = 0.735), the times of entry into the open arms (*t*-test, *t* = 0.82, *P* = 0.425), and the total distances travelled (*t*-test, *t* = 0.99, *P* = 0.343) between the two groups (*n* = 8 per group). (e) Schema of the open field and the representative activity traces during an open field test in control mice and mice with 1-EBIO injection. (f–h) There was an insignificant difference in the duration in central area (*t*-test, *t* = 0.36, *P* = 0.725), the courses in the central area (*t*-test, *t* = 0.17, *P* = 0.866), and the total distances travelled (*t*-test, *t* = 1.15, *P* = 0.272) between the two groups (*n* = 8 per group). (i) Schematic diagram of the sucrose preference test. (j) The percentage of the sucrose intake volume over the total fluid intake volume in mice with 1-EBIO injection was consistent with that in control mice (*t*-test, *t* = 0.03, *P* = 0.975, *n* = 8 per group). (k) Schematic representation of mobility and immobility in the forced swim test. (l) The percentage of immobility duration over the total time in mice with 1-EBIO injection was consistent with that in the control mice (*t*-test, *t* = 0.02, *P* = 0.984, *n* = 8 per group). Data are expressed as mean ± SEM.

Mice underwent intrathecal administration of 1-EBIO (30 *μ*g/5 *μ*L) 2 h prior to behavioral tests. Similar to apamin injection, 1-EBIO administration did not affect the anxiety-like behavior of mice. The duration spent in the open arms (*n* = 8, *P* = 0.735, Figures 7(a) and 7(b)), the times of entry into the open arms (*n* = 8, *P* = 0.425, Figures 7(a) and 7(c)), the duration in central area (*n* = 8, *P* = 0.725, Figures 7(e) and 7(f)), and the courses in central area (*n* = 8, *P* = 0.866, Figures 7(e) and 7(g)) showed insignificant differences between the control mice and mice with 1-EBIO injection. Similarly, 1-EBIO administration did not affect the depression-like behavior as evidenced by the same preference for sucrose (*n* = 8, *P* = 0.975, Figures 7(i) and 7(j)) and immobility duration (*n* = 8, *P* = 0.984, Figures 7(k) and 7(l)) between the two groups.

### 3.8. Contribution of Downregulated MPP2 to the Downregulation of Membrane SK2 Protein

**Figure 8:**
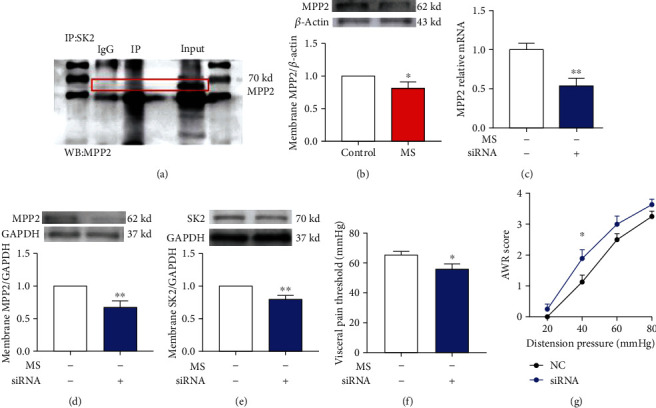
The downregulation of MPP2 contributed to the decrease in the membrane SK2 channel protein and visceral pain threshold. (a) From western blotting of spinal cord cell lysates expressing anti-MPP2 antibody and immunoprecipitation with SK2 or IgG (control), adjacent blotting showed input of MPP2. (b) Spinal MPP2 protein in the membrane fraction presented a significant decrease in mice experiencing neonatal MS versus the control mice (*t*-test, *t* = 2.25, *P* < 0.05, *n* = 6 per group). (c) Spinal MPP2-related mRNA presented a significant decrease following the siRNA targeting MPP2 administration (*t*-test, *t* = 3.56, *P* < 0.01, *n* = 6 per group). (d) The siRNA targeting MPP2 injection resulted in the decrease of spinal membrane MPP2 protein in naïve mice (*t*-test, *t* = 3.59, *P* < 0.01, *n* = 6 per group). (e) The siRNA targeting MPP2 administration led to the decrease of spinal SK2 channel in the membrane fraction (*t*-test, *t* = 3.54, *P* < 0.01, *n* = 6 per group). (f) The visceral pain threshold in naïve mice presented a significant decrease after injection of the siRNA targeting MPP2 (*t*-test, *t* = 2.21, *P* < 0.05, *n* = 8 per group). (g) The AWR score presented a significant increase in naïve mice subjected to pressure stimulus at 40 mmHg (Bonferroni *post hoc* test, *P* < 0.05) after injection of the siRNA targeting MPP2 (two-way ANOVA test, *F* (1, 14) = 3.76, *P* = 0.07, *n* = 8 per group). Data are expressed as mean ± SEM; ^∗^*P* < 0.05 and ^∗∗^*P* < 0.01.

To further explore the mechanism of SK2 downregulation in MS mice, we focused on MPP2, which is a synaptic scaffold protein located in postsynaptic density (PSD), and SK2 acts by anchorage on the synaptic membrane via MPP2. As depicted in Figure 8(a), with coimmunoprecipitation, we identified that SK2 interacted with MPP2. Western blotting data revealed a significant decrease of the spinal MPP2 protein in the membrane fraction in mice experiencing neonatal MS (*n* = 6, *P* < 0.05, Figure 8(b)). In addition, qRT-PCR, western blotting data, and behavioral tests further confirmed our speculation that MPP2 downregulation contributed to the decrease in the membrane SK2 protein. The naïve mice were injected with either siRNA of MPP2 or the negative control (NC) of siRNA, and the aforementioned tests were conducted 30 h thereafter. qRT-PCR data showed that spinal MPP2-related mRNA presented a significant decrease following the siRNA targeting MPP2 administration (*n* = 6, *P* < 0.01, Figure 8(c)). Consistently, the same was also true of the membrane MPP2 (*n* = 6, *P* < 0.01, Figure 8(d)) and SK2 protein in membrane fraction (*n* = 6, *P* < 0.01, Figure 8(e)). The visceral pain threshold in naïve mice presented a significant decrease following injection of the siRNA targeting MPP2 (*n* = 8, *P* < 0.05, Figure 8(f)). Consistently, after injection of the siRNA targeting MPP2 in naïve mice, the AWR score was significantly increased with pressure stimulus at 40 mmHg (*n* = 8, *P* < 0.05, Figure 8(g)). Taken together, these results suggested that the downregulation of MPP2 may serve to explicate the downregulated membrane SK2 protein in mice experiencing neonatal MS.

## 4. Discussion

SK channels in the spinal DH play vital roles in nociception modulation [34–36]. In this study, we investigated the role of SK2 channels in the spinal DH in visceral hypersensitivity induced by maternal separation. The present findings provided evidence that mice experiencing neonatal MS were susceptible to visceral stimuli in adulthood, along with significant downregulation of the spinal membrane SK2 channel protein and SK2-mediated *I*_AHP_, and an increase in neuronal firing rates in the spinal DH. Application of SK2 channel blocker apamin could exacerbate visceral hypersensitivity by reducing *I*_AHP_ and increasing neuronal firing rates in naïve mice. Intrathecal injection of SK2 channel activator 1-EBIO alleviated visceral hypersensitivity and reversed the alteration of *I*_AHP_ and neuronal firing rates in mice experiencing MS. MPP2 could interact with SK2, accompanied by a downregulation of the membrane MPP2 protein expression in mice exposed to MS. In addition, disruption of MPP2 by siRNA aggravated visceral pain in naïve mice. These findings were in line with prior evidence that SK2 channels in the spinal DH are involved in visceral hypersensitivity induced by ELS [42].

Adverse experiences in early life can affect the formation of neuronal circuits and exert long-term effects on neuronal function, which is deemed as a potential risk factor of increased physical and psychological morbidity in adulthood [43]. Accumulating evidence has authenticated that ELS can lead to corresponding abnormalities of learning and memory as well as anxiety- and depression-like behaviors [9, 10]. These reports are supportive of our finding that mice exposed to MS exhibited anxiety- and depression-like behaviors. The anxiety-like behavior tests in our study included open field test and elevated plus maze test in parallel with sucrose preference test and forced swimming test to verify depression-like behaviors. Moreover, the stressor patterns can be divided into two major categories: physical (which actually disturbs physiological state) and psychological (which threatens the current or expected status) [18, 19]. Early physical and psychological life stresses can lead to different long-term behavioral and physical alterations by activation of diverse neural networks [16–18], both contributing to changes of neuroendocrine and signal pathways involved in regulating neuroplasticity. In addition, behavioral abnormalities include somatic and visceral hyperalgesia [11, 12, 44, 45]. Our findings were in line with these studies that mice experiencing MS developed visceral hypersensitivity.

As is well acknowledged, visceral information originating from the distal colon and rectum can be converged to the thoracolumbar and sacral segments of the spinal cord, whereas a number of thoracolumbar spinal neurons can also receive input from afferents of the superficial skin and deep somatic domain [46]. Accordingly, the spinal cord is highly subjected to modulation of somatic and visceral sensory afferents. Structural and functional abnormalities of spinal DH neurons are involved in inflammatory pain, neuropathic pain, and other pain disorders [47, 48]. Moreover, the visceral nociceptive neurons in the spinal DH reportedly exhibit hyperexcitability in animal subjects with visceral hypersensitivity [24–26], which invites further exploration of the mechanism of spinal DH involved in visceral hypersensitivity.

In this study, we adopted maternal separation to establish the animal model of visceral hypersensitivity. Separation of pups from their dams was conducted daily for 6 h as of postnatal day 2 to15, with distinction from studies of MS models established by separating pups from their dams 3 h daily. As per their findings, separation for 3 h daily can develop somatic and visceral hyperalgesia as well [49–52]. Nevertheless, we have recognized that exposure to MS 3 h daily could not develop visceral hypersensitivity in adulthood, whereas separation for 6 h daily could successfully induce visceral hypersensitivity, which was contrary to the abovementioned results [51, 52] and consistent with our previous reports [53]. We attributed this discrepancy to the distinction in time course of separation and animal species.

Our data revealed that mice exposed to maternal separation presented a significant expression downregulation of the spinal membrane SK2 channel protein and SK2-mediated *I*_AHP_. Synaptic SK2-containing channels can modulate the induction of synaptic plasticity and excitatory postsynaptic responses, due to the activation by synaptically evoked Ca^2+^ influx [38]. Our study indicated that the downregulation of the membrane SK2 channel protein expression contributed to an elevation of neuronal excitability which was validated by an increase in neuronal firing rates in spinal DH. The SK channels mediate medium *I*_AHP_ conductances in neurons across the central nervous system, which is involved in the regulation of neuronal firing, domination of LTP, and modulation of memory activities [54]. Moreover, the enhancement of the SK2 channel function in the spinal cord can attenuate the thermal stress-induced nociceptive behavior by reducing spike discharges and increasing *I*_AHP_ amplitudes [34]. In contrast, intrathecal injection of specific SK channel inhibitor apamin can lead to SK2-mediated *I*_AHP_ downregulation with a decrease in visceral pain threshold in mice experiencing colorectal distension [42].

In line with prior reports, our data confirmed that SK2 channel activator 1-EBIO could prevent the development of visceral hypersensitivity and reverse the changes of *I*_AHP_ and neuronal firing rates in mice subjected to MS; SK2 channel blocker apamin induced visceral hyperalgesia by reducing *I*_AHP_ and increasing neuronal firing rates in naïve mice. In addition, western blotting results revealed that intrathecal injection of 1-EBIO or apamin could result in an increase or a decrease in the membrane SK2 channel protein expression in spinal DH, which further supports the notion that the function of membrane SK2 channels is closely associated with neuronal excitability, whereby exerting effects on visceral hypersensitivity. Similar to SK2 channels, high levels of SK3 channels are identified in the spinal DH, particularly in laminae I and II [31]. However, our previous study of the SK3 channel in spinal DH vetoed the involvement of the SK3 channel in visceral hypersensitivity [42]. On this ground, we excluded the SK3 channel as our research target in this study.

Moreover, since MS could induce anxiety- and depression-like behaviors, it would be of interest whether modulation of the membrane SK2 expression in the spinal DH could lead to the alteration of these behavior phenotypes. Paradoxically, our findings indicated that the SK2 channel in the spinal DH was not involved in the modulation of anxiety- and depression-like behaviors. However, it has been reported that SK2 channel overexpression in basolateral amygdala-projecting neurons prevents stress-induced anxiety-like behavior [55]. In addition, ELS can induce anxiety- and depression-like behaviors via dysregulation of the neuroendocrine system which includes the HPA axis and the serotonergic system, along with changes of brain-derived neurotrophic factor expression [56–59]. Overactivation of the immune system can also lead to the abnormalities in anxiety- and depression-like behaviors [60]. We attributed this discrepancy in the behavior to the distinction of designation of modulation targets, which invites our future exploration.

Given the absence of significant differences in the expression of SK2 mRNA and total SK2 channel protein in the spinal DH in mice experiencing MS versus the control group and the presence of significant difference in the membrane SK2 channel protein expression in MS model mice, we further explored the molecular mechanism of the synaptic localization of the SK2 channel in the spinal DH. MPP2 is a novel synaptic scaffold, which is localized in postsynaptic sites in hippocampal neurons by binding to the abundant postsynaptic scaffold proteins such as PSD-95 and guanylate kinase-associated protein (GKAP) [61]. Moreover, MPP2 has been recently demonstrated to be responsible for proper synaptic localization and function of SK2-containing channels in hippocampal CA1 pyramidal neurons [38]. In our study, coimmunoprecipitation findings affirmed that MPP2 could interact with SK2 in the spinal cord DH, along with a decrease in the membrane MPP2 protein expression. Furthermore, disruption of the MPP2 expression by intrathecal injection of siRNA exacerbated visceral hypersensitivity in MS model mice, which further supported the role of MPP2 involved in visceral hypersensitivity. However, there are reports that synaptic membrane-associated guanylate kinase (MAGUK) proteins can influence N-methyl-D-aspartate receptor- (NMDAR-) mediated synaptic function and associated persistence of pain by regulating surface and synaptic NMDAR trafficking at the spinal cord level [62–64]. Therefore, the interactions between MPP2 and SK2 in the spinal DH in the modulation of visceral hypersensitivity await further exploration.

## 5. Conclusions

In conclusion, we explored the contribution of SK2 channels in the spinal DH to visceral hypersensitivity in mice. Our research has demonstrated that neonatal MS leads to visceral hypersensitivity via the downregulation of SK2 channels. Pharmacological activation of SK2 channels can prevent the precipitation of visceral hypersensitivity, and blockade of SK2 channels can aggravate visceral hypersensitivity. Moreover, the downregulation of MPP2 may underlie the decrease in membrane SK2 channels. This study may unveil one potential pathogenesis of IBS and may provide a novel avenue to the development of therapeutic agents for IBS and visceral hypersensitivity with efficiency and efficacy.

## Data Availability

Raw data were generated with the use of the ImageJ software (NIH, USA), Clampex and Clampfit 10 (Axon Instruments, San Jose, CA, USA), etc. Derived data to support the findings of this study are available from the corresponding author upon request.
